# An Infrared Study of Gas-Phase Metal Nitrosyl Ion–Molecule Complexes

**DOI:** 10.1021/acs.jpca.2c07228

**Published:** 2022-12-08

**Authors:** Gabriele Meizyte, Philip A. J. Pearcy, Peter D. Watson, Edward I. Brewer, Alice E. Green, Matthew Doll, Olga A. Duda, Stuart R. Mackenzie

**Affiliations:** Department of Chemistry, University of Oxford, Physical and Theoretical Chemistry Laboratory, South Parks Road, Oxford, United Kingdom, OX1 3QZ

## Abstract

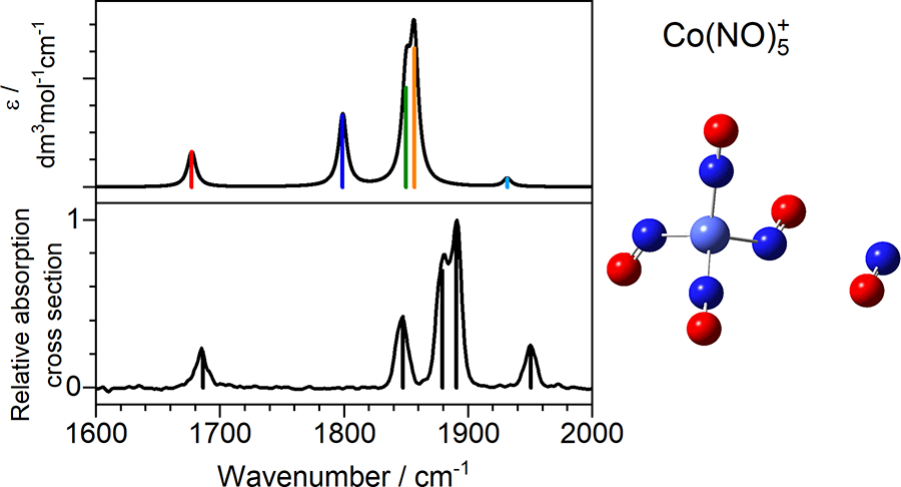

We present a combined experimental and quantum chemical study of gas-phase group 9 metal nitrosyl complexes, M(NO)_*n*_^+^ (M = Co, Rh, Ir). Experimental infrared photodissociation spectra of mass-selected ion-molecule complexes are presented in the region 1600 cm^–1^ to 2000 cm^–1^ which includes the NO stretch. These are interpreted by comparison with the simulated spectra of energetically low-lying structures calculated using density functional theory. A mix of linear and nonlinear ligand binding is observed, often within the same complex, and clear evidence of coordination shell closing is observed at *n* = 4 for Co(NO)_*n*_^+^ and Ir(NO)_*n*_^+^. Calculations of Rh(NO)_*n*_^+^ complexes suggest additional low-lying five-coordinate structures. In all cases, once a second coordination shell is occupied, new spectral features appear which are assigned to (NO)_2_ dimer moieties. Further evidence of such motifs comes from differences in the spectra recorded in the dissociation channels corresponding to single and double ligand loss.

## 1 Introduction

Nitrogen oxides (NO_*x*_) are well-known for their detrimental effects on the environment and as a major source of air pollution harmful to human health.^1,2^ Although formed naturally, e.g., in lightning and/or forest fires, anthropogenic sources represent a significant fraction of NO_*x*_ production. Both NO_2_ and NO are generated by the internal combustion engine, and this has led to the introduction of stringent emission controls.^3,4^ Current mitigation strategies include the widespread use of automobile catalytic converters which harness surface catalytic chemistry of finely dispersed transition metals.^5,6^ In turn, this has stimulated extensive research aimed at developing a better understanding of this chemistry including that of nitric oxide, NO, the subject of this work. For its role in both the environment and in biology, NO was named *Science* magazine’s molecule of year for 1992^7^ and a whole edition of *Chemical Reviews* was dedicated to NO chemistry.^8^

Atomic ions and small gas-phase metal clusters can represent tractable model systems, providing molecular level insight into fundamental metal–ligand interactions, including those important in catalytic chemistry, free of complications arising from substrates, solvation, or aggregation.^9^ The thermal reactivity of NO with a wide range of gas-phase metal cations has been studied by Bohme and co-workers by inductively coupled plasma/selected-ion flow tube (ICP/SIFT) tandem mass spectrometry.^10^ This revealed extensive and varied chemistry involving electron donation and both atom acceptor and donor reactions. Relevant to the metal centers studied here, a mix of molecular and dissociative NO binding on Co_*n*_^+^ clusters has been observed under different conditions.^11−14^ Rh_*n*_^±^ reactions with NO have been studied extensively under single^15−17^ and multiple collision conditions, and the chemistry has been reviewed recently.^18^ There have also been spectroscopic studies of NO binding to Au_*n*_^+^ and Ir_*n*_^+^ clusters.^19,20^

NO is an intriguing ligand in coordination chemistry. A kinetically inert, stable radical, it exhibits a range of different binding motifs, coordinating formally via one- or three-electron donation, and nitrosyl complexes often exhibit very different chemistry to their carbonyl analogues.^21^ The open-shell nature of nitric oxide presents particular challenges for quantum chemical calculations. Blanchet et al. employed density functional theory (DFT) to study first row transition metal atom-NO molecules. They established nonlinear binding through the N atom in CoNO arising from the Co 3d and 4s orbitals with the highest occupied 5σ orbital in NO with a triplet ground state favored due to symmetry breaking.^22^ Bauschlicher and Hall applied DFT and coupled cluster calculations to the equivalent cationic species and, for CoNO^+^, found a linear geometry preferred.^23^

This work presents an infrared photodissociation (IR-PD) study of NO binding to group 9 cations Co(NO)_*n*_^+^, Rh(NO)_*n*_^+^, and Ir(NO)_*n*_^+^ (*n* = 3–7) with interpretation aided by comparison with simulated spectra of low-energy structures calculated by DFT. The present work follows detailed IR-PD studies of M(NO)_*n*_^+^ (M = Fe, Cu, Ag, and Au) complexes by Zhou and co-workers which showed marked differences in the structures adopted for different metal ions.^24−26^ Fe(NO)_*n*_^+^ structures exhibit linear (three-electron) binding until closure of the first coordination shell is achieved at 4, with the messenger Ar tag atoms leading to strong distortions.^24^ Cu(NO)_*n*_^+^ complexes are characterized by bent (one-electron) binding and significant ligand–ligand interactions with clear evidence of bidentate dimer (NO)_2_ ligand.^25^ Nonlinear ligand binding and dimer structures were also observed in Ag(NO)_*n*_^+^ and Au(NO)_*n*_^+^ complexes albeit with first coordination shells of 4 and 2, respectively.^26^

## 2 Experimental and Computational Methods

The experimental setup has been described in detail previously^27,28^ and modified recently to include the mass selection of ions via a quadrupole mass filter.^29^ Briefly, a rotating disc target of the required metal (Co, Rh, or Ir) is ablated at 532 nm by a pulsed Nd:YAG laser (10 Hz, ca. 2–10 mJ as necessary). Ablated species are entrained in a gas pulse of Ar seeded with 1–5% NO which expands into the vacuum forming a molecular beam with the higher NO mole fractions required for efficient generation of Ir(NO)_*n*_^+^. The beam is skimmed before passing through a quadrupole mass filter–quadrupole bender assembly and then entering the extraction region of a reflectron time-of-flight (ToF) mass spectrometer.

To record IR-PD spectra, the cluster beam is subjected to tunable pulsed infrared radiation from a tabletop optical parametric oscillator/optical parametric amplifier (OPO/OPA, Laservision) system, operating in the 1600–2000 cm^–1^ range. Following mass-selection of the parent ion–molecule complex, spectra are recorded as a function of wavenumber in the daughter fragment channels against a zero background.

To account for variations in the infrared pulse energy across the wavenumber region scanned, spectra are reported here in terms of a normalized cross-section in a given fragment channel using a modified Beer–Lambert law:^30^
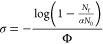
1in which σ is the absorption cross-section, *N*_f_ and *N*_0_ correspond to the intensity of the fragment and parent signals, respectively, and Φ is the photon flux. The factor α describes the overlap between the ion beam, and the infrared beam and is assumed to be 1. Spectra are normalized to the strongest peak in the spectrum.

To aid assignment, experimental M(NO)_*n*_^+^ IR-PD spectra are compared with simulated spectra of energetically low-lying structures determined from DFT using the Gaussian 16 software package^31^ with novel potential structures generated by a modified KICK algorithm.^32^ It is worth noting that open-shell ligands such as NO pose particular difficulties for DFT, as complexes often give rise to multiple low-lying electronic states as well as large numbers of isomeric forms.^33^ The B3P86^34,35^/def2TZVP^36,37^ functional/basis set combination was used throughout, having proven reliable in similar studies by our group.^38,39^ To provide a better match with experimental data, computed harmonic vibrational frequencies were scaled by a factor of 0.9347, determined by calculating the vibrational frequency of the free NO stretch which is found experimentally at 1876 cm^–1^.^40^ Calculated spectral lines are convoluted with Lorentzian line shapes with full width half-maximum (fwhm) = 8 cm^–1^ to aid comparison with spectra. All energies reported here include zero-point energy corrections. Only molecularly bound ligands have been considered, as these are the only structures expected to exhibit spectra in our experimental region. Dissociatively bound complexes lie substantially lower in energy, but access to these minima is kinetically hindered by large activation barriers.

## 3 Results and Discussion

### A Time-of-Flight Mass Spectra

**Figure 1:**
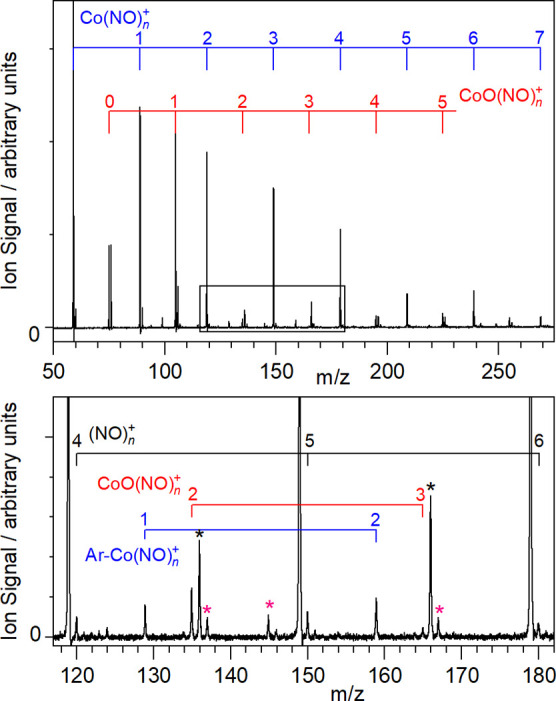
Time-of-flight mass spectrum produced upon ablating the Co metal target in the presence of NO in Ar gas mix (1.5%). The lower panel shows an expanded region indicated in the top panel. Other species observed include [O(NO)_*n*_]^+^ complexes (black asterisks), Ar-tagged peaks and water-containing species (red asterisks).

A typical time-of-flight mass spectrum produced by laser ablation of a Co target in the presence of NO seeded in Ar is given in Figure 1. The mass spectrum is dominated by the Co(NO)_*n*_^+^ peaks. Smaller mass species are favored under expansion conditions used here, with a marked drop in signal intensity for *n* > 4 which is suggestive of a first coordination sphere of four ligands. Consistent with the reactivity study of Bohme and co-workers, CoO^+^ and CoONO^+^ are also formed in significant number densities^10^ along with several Ar-tagged species.

Mass spectra obtained following Rh and Ir ablation can be found in Supporting Information. The rhodium mass spectrum is cleaner than that for cobalt and is dominated by the progression of Rh(NO)_*n*_^+^ (*n* = 0–7) peaks whose intensity decrease monotonically with *n* with little obvious sign of clear coordination shells. Of the oxides, only [RhO(NO)^+^] is formed in significant number density. Iridium has two stable isotopes of 191 u and 193 u, and this complicates the mass spectrum. Even so, the mass spectrum is markedly richer than the cobalt and rhodium. Below *m*/*z* = 300 the mass spectrum is dominated by strong Ir(NO)_*n*_^+^ and IrO(NO)_*n*_^+^ signals, the latter reflecting the O atom transfer reactivity of Ir^+^ toward nitric oxide.^10^ Perhaps as a result of this reactivity it proved impossible to generate Ir(NO)_*n*_^+^ (*n* > 5) in sufficient number density to record infrared spectra with acceptable signal-to-noise ratio.

In all mass spectra there is evidence for naked (NO)_*n*_^+^ and NO_2_(NO)_*n*_^+^ clusters. These are produced within the ablation plasma, and their infrared action spectra will be the subject of a future study.

### B Infrared Spectra of M(NO)_*n*_^+^ Ion–Molecule Complexes

**Figure 2:**
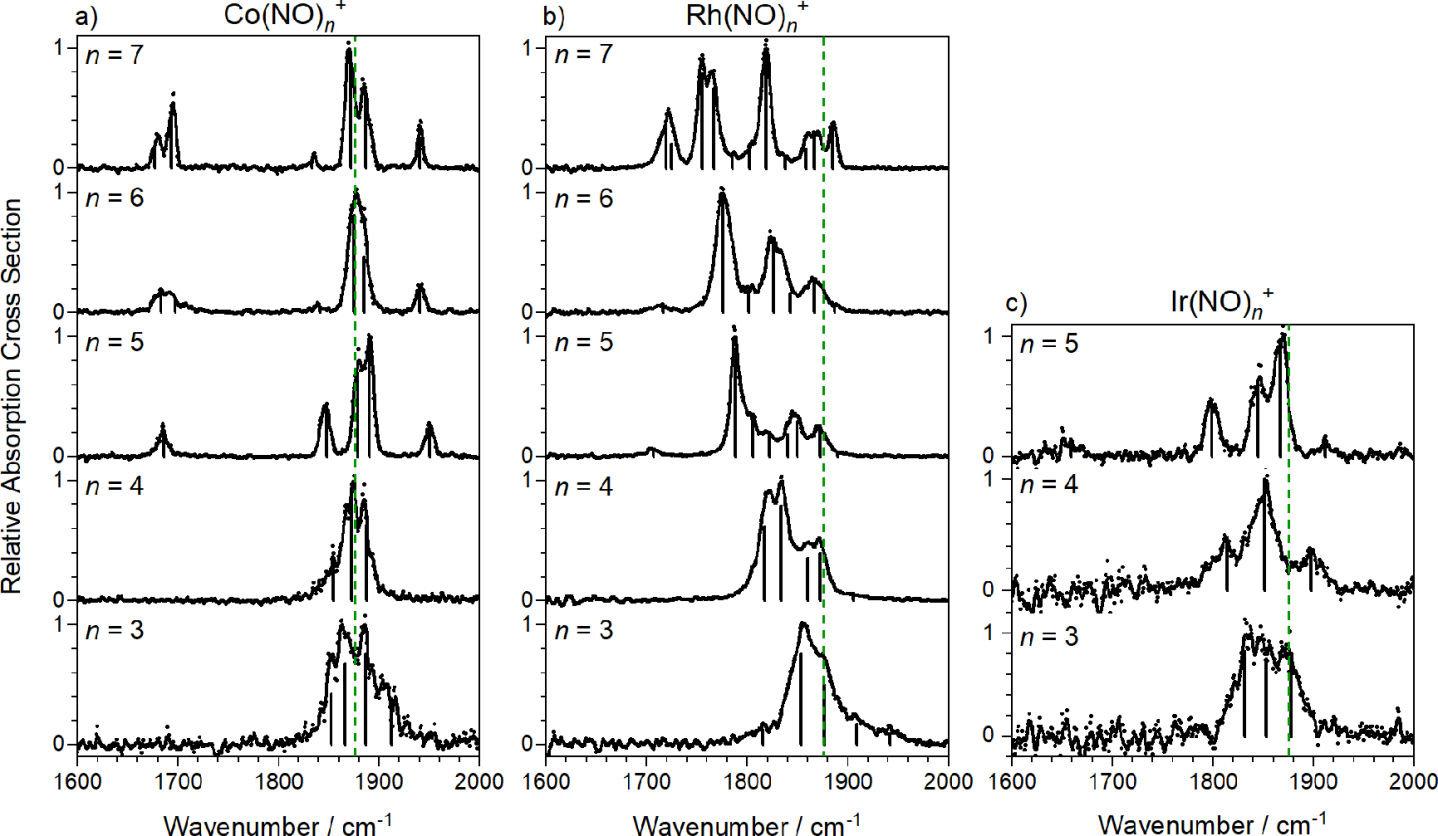
Infrared photodissociation spectra of (a) Co(NO)_*n*_^+^, (b) Rh(NO)_*n*_^+^, and (c) Ir(NO)_*n*_^+^ complexes, recorded in the enhancement of the M(NO)_*n*−1_^+^ daughter channel and shown as normalized relative absorption cross-sections for each complex size. Vertical lines arise from fitting of the spectrum to Gaussian functions. Green dashed lines indicate the wavenumber of the free NO stretch at 1876 cm^–1^.^41^

An overview of the infrared action spectra of M(NO)_*n*_^+^ (M = Co, Rh, Ir) complexes is shown in Figure 2. In all cases the dominant fragmentation observed following photoexcitation is NO loss, and the spectra shown are recorded against a zero background in enhancement in the daughter M(NO)^+^_*n*–1_ (i.e., simple NO loss) mass channel. Vertical lines shown under each spectrum represent the centers of Gaussian functions used to fit to the spectra from which trends in band positions can be extracted as well as providing an estimate of the number of bands contributing. For each spectrum, the full width at half-maximum was fixed to that of the most highly resolved band and the number of bands was guided by the number of infrared active modes in simulated spectra.

Negligible fragmentation was observed for the very smallest, *n* = 1, 2 complexes. This is common in IR-PD and reflects the fact that the first few ligands typically bind strongly to a metal center and that many photons are required to dissociate the complex. In the present case, the binding energies of each of the first two nitrosyl ligands is ≥1.4 eV (see Supporting Information), while the photon energy in this spectral region is ca. 0.20–0.25 eV (of course, the complexes have significant internal energy of their own). For similar reasons, the spectra of the *n* = 3, 4 complexes exhibit comparatively poor signal-to-noise ratios, reflecting their own weak fragmentation efficiency. Their broad, partially resolved appearance suggests that in these cases, too, multiple photons are needed to drive NO loss. By contrast, the infrared spectra of the larger M(NO)_*n*_^+^ (M= Co, Rh, Ir, *n* ≥ 5) complexes are both significantly better resolved and exhibit higher signal-to-noise ratios. This is consistent with the binding energy of the fifth (and higher) NO ligand being comparable with the IR photon energy (see Supporting Information).

The evolution of the Co(NO)_*n*_^+^ and Ir(NO)_*n*_^+^ spectra with cluster size is similar with most intensity observed close to the free NO stretch at 1876 cm^–1^ and new features appearing for larger complexes. In these regards the spectra are very similar to those reported previously for Fe(NO)_*n*_^+^.^24^ The Rh(NO)_*n*_^+^ spectra, by contrast, are qualitatively different, and although there is a persistent band around the free NO stretch, a noticeable overall red shift in the IR spectrum is observed with increasing *n* and any satellite bands are much less prominent.

In the spectra of Co(NO)_*n*_^+^ complexes, a new pair of bands appears for larger (*n* ≥ 5) complexes with one band around 1680 cm^–1^ and another near 1950 cm^–1^. Similar pairs of bands are visible in the Ir(NO)_5_^+^ spectrum and, to a lesser extent, the Rh(NO)_*n*_^*+*^ (*n* > 4) spectra. Zhou and co-workers have also reported very similar bands in the spectra of M(NO)_*n*_^+^ (M = Au, Ag, Cu, Fe). These bands have been attributed to the formation of a dimer, (NO)_2_, motif forming following closure of the inner coordination shell.^24−26^ The appearance of these bands is thus consistent with an inner coordination shell of four with the fifth ligand forced to start a new coordination shell and binding preferentially to one of the inner-shell ligands.

### C Comparison of Experimental and Simulated Spectra

The binding of the NO radical at charged metal centers is a complex balance of many contributing factors. The dominant electrostatic interaction is the charge–dipole attraction which favors N-binding with the proximity of the metal cation strongly polarizing the NO bond. In addition, there are multiple components of incipient chemical bonding arising from (i) NO lone-pair σ donation, (ii) π donation from the unpaired NO π* electron, and (iii) π-back bonding from the occupied d orbitals on the metal center into the NO π* orbitals. The relative contributions of these factors lead to subtle structural differences which are reflected in the infrared spectra. For example, σ-donation, in which NO acts formally as a three-electron donor, leads to near linear M^+^–N=O binding. By contrast, one-electron π*-donation leads to nonlinear coordination.^42^ The latter was found to dominate the structures of the coinage-metal nitrosyl complexes^25,26^ while a mix of the two was invoked to interpret the spectra of Fe(NO)_*n*_^+^.^24^

**Figure 3:**
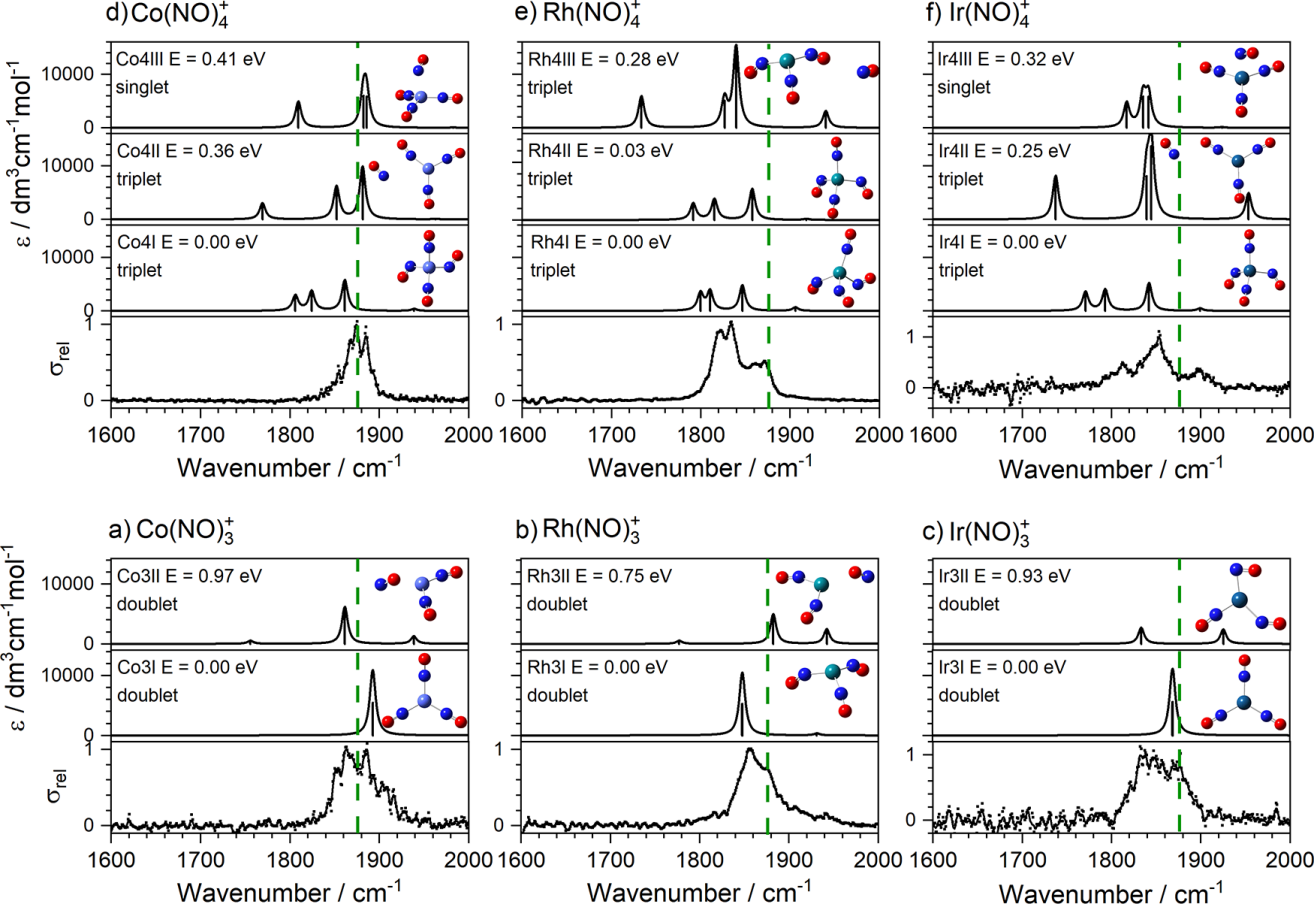
Comparison of experimental and simulated spectra for M(NO)_*n*_^+^ (M = Co, Rh, Ir; *n* = 3 (a–c), 4 (d–f)) complexes showing the lowest energy calculated structures only. Green dashed lines indicate the wavenumber of the free NO stretch at 1876 cm^–1^.^40^

Figure 3 shows a comparison of experimental and simulated spectra for the M(NO)_*n*_^+^ (M = Co, Rh, Ir, *n* = 3, 4) complexes. Extended versions of this figure with additional (higher-lying) calculated structures and electronic states are provided in Supporting Information. Notwithstanding the broad, unresolved nature of the experimental spectra, a few clear conclusions can be drawn. For *n* = 3 complexes, all low energy structures (within the lowest 1 eV) have doublet multiplicity. A clear lowest energy isomer is identified, labeled M3I (M = Co, Rh, Ir), whose simulated spectrum accounts well for that observed. Co3I and Ir3I are planar *D*_3*h*_ structures with rigidly linear ligand binding indicating the dominance of (three-electron) σ-donation. By contrast, the bent *C*_3*v*_ Rh3I reflects π-donation. There is negligible evidence of ligand activation in either the wavenumber of the spectral bands or the calculated NO bond lengths. In all cases ligands bind via the nitrogen atom with, at most, weak evidence for the presence of energetically higher-lying O-bound structures.

The lowest energy M(NO)_4_^+^ structures are all triplet multiplicity, tetrahedral-based, N-bound structures. We calculate low-lying excited electronic states for each species (see Supporting Information) as well as multiple isomeric structures on the ground-state surface, some of which include a second coordination shell (e.g., Co4II, Rh4III, and Ir4II). There is, however, little evidence for these structures in the experimental spectra. In both Co(NO)_4_^+^ and Ir(NO)_4_^+^ the lowest energy structures identified (Co4I and Ir4I) have two linear and two nonlinear bound ligands. Again, though, the effect on the bond lengths is negligible. The equivalent Rh(NO)_4_^+^ structure (Rh4II) lies just 0.03 eV higher than the lowest energy Rh4I structure, with three of its ligands bound nonlinearly. There is little in the experimental spectrum to distinguish between them, and it is possible that both are present.

**Figure 4:**
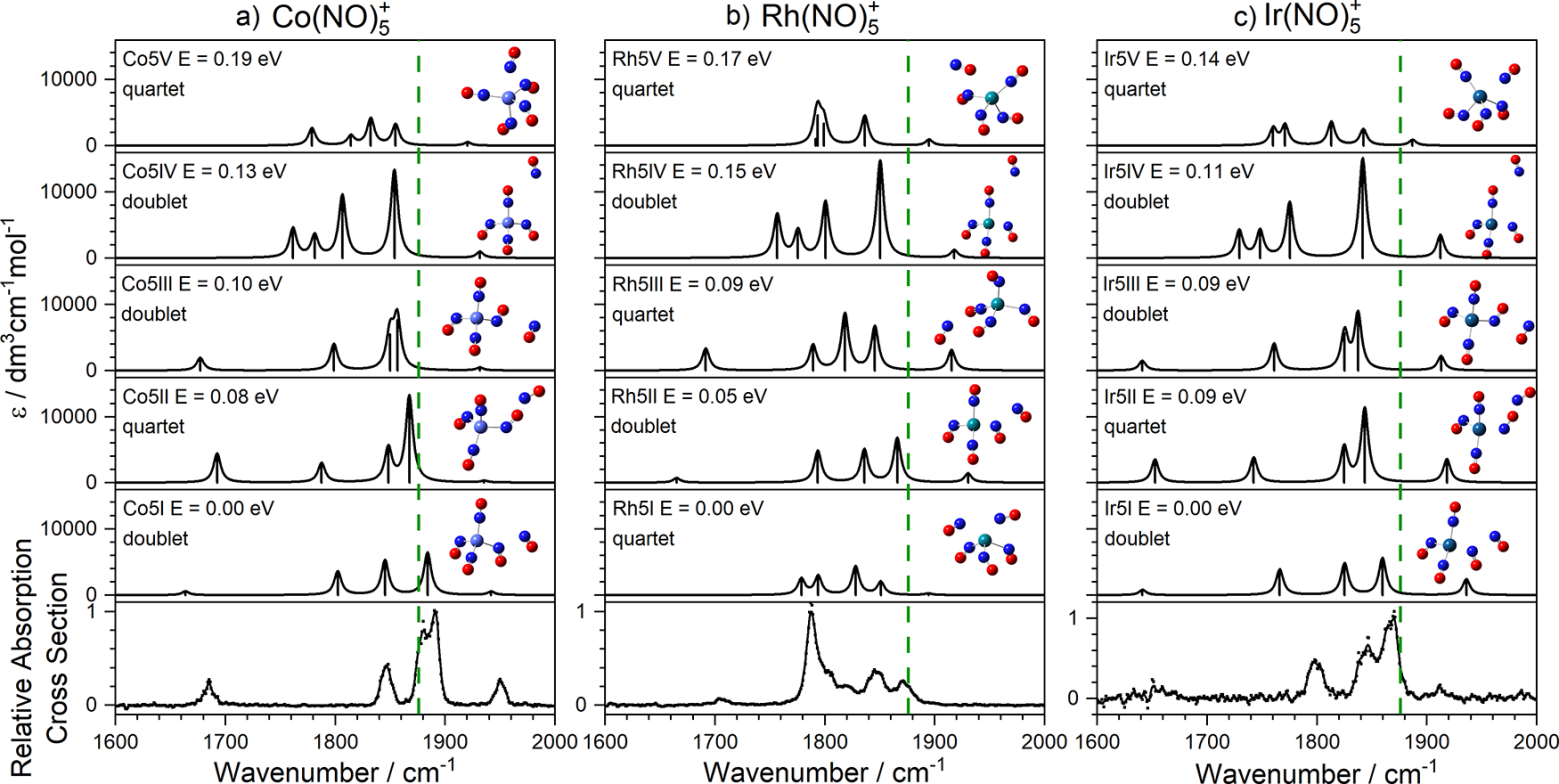
Comparison of experimental and simulated spectra of low-lying isomers for (a) Co(NO)_5_^+^, (b) Rh(NO)_5_^+^, and (c) Ir(NO)_5_^+^. In most cases the fifth ligand binds more weakly in a second coordination shell giving rise to new spectral features. Green dashed lines indicate the wavenumber of the free NO stretch at 1876 cm^–1^.^40^

With the addition of a fifth ligand, our calculations show the opening of a second coordination shell and binding in dimer motif to one of the inner ligands (Figure 4). The exception is the lowest energy calculated structure for Rh(NO)_5_^+^ (structure Rh5I) which shows a five-coordinate structure. The weaker binding in the second shell gives rise to a richer distribution of both low-lying electronic states (doublet and quartet states) and structural isomers predicted as shown in Figure 4. In all cases, the signature of the (NO)_2_ motif is provided by the new vibrational bands, most prominent in the spectrum of Co(NO)_5_^+^ at 1680 and 1950 cm^–1^ but also visible in the other two complexes.

**Figure 5:**
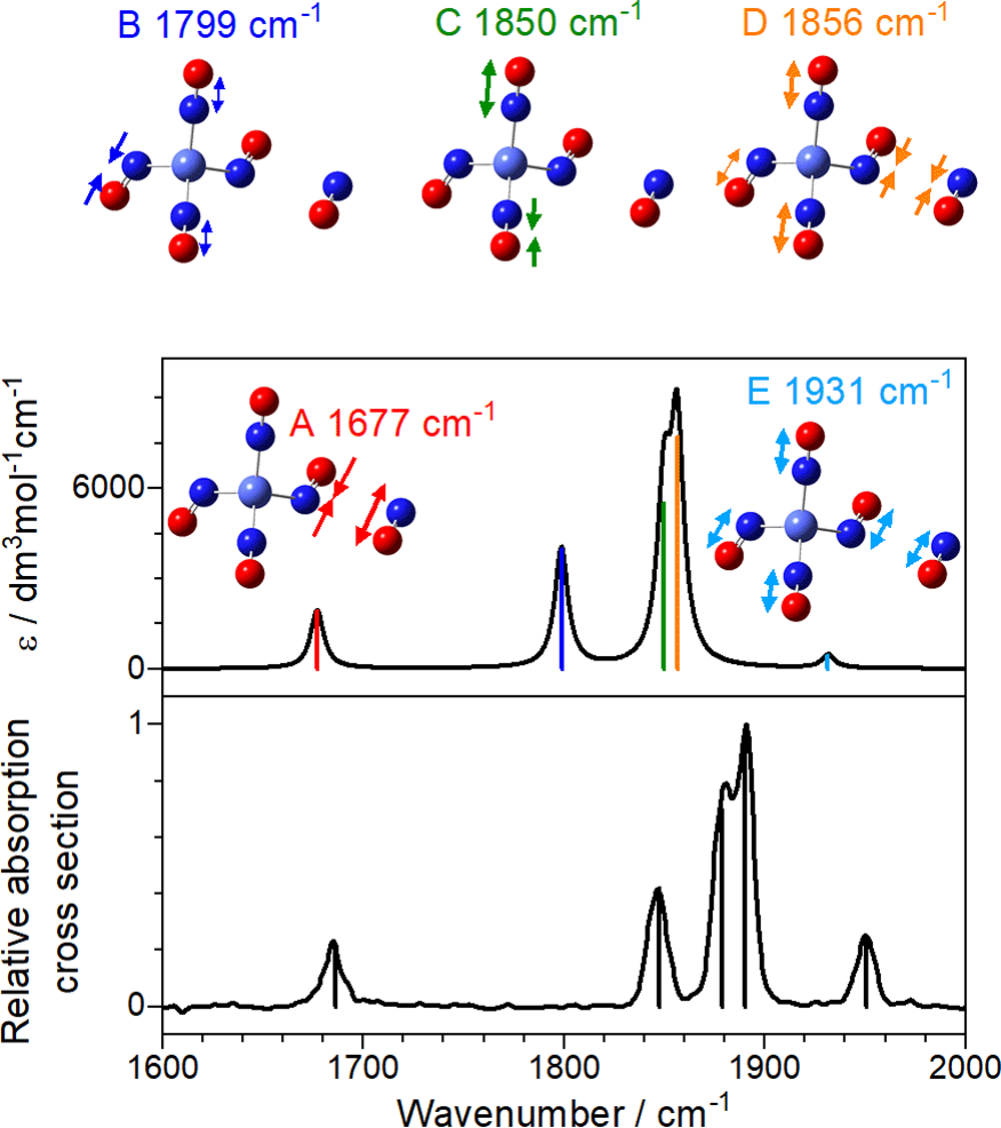
A comparison of the experimental and computational spectrum (Co5III) for Co(NO)_5_^+^ with color-coded mode vectors.

In the case of Co(NO)_5_^+^ and Ir(NO)_5_^+^ there is convincing agreement between the experimental spectrum and that of the energetically low-lying structures. Figure 5 shows one such comparison for the Co(NO)_5_^+^ spectrum with the simulated spectrum of structure Co5III (*E* = 0.1 eV above the lowest energy calculated structure). This structure exhibits an NO-dimer in trans form though the isomerism of the dimer has very minor effect on the spectrum. Figure 5 also depicts the ligand vibrations responsible for the five high frequency infrared active bands in this region (labeled A–E). The A mode at ca. 1680 cm^–1^ is highly localized and arises from the antisymmetric motion of the two ligands comprising the dimer moiety. The red-shift in this band reflects weak activation of the inner of the two dimer ligands whose bond length (1.146 Å) is slightly longer than that of its partner (1.119 Å). The B mode comprises out-of-phase vibrations of the three nondimer ligands, mainly that opposite the dimer. The C mode (calculated at 1850 cm^–1^, observed at 1880 cm^–1^) involves out-of-phase motion of the two near linear bound ligands. Finally, the two highest frequency modes, D and E, comprise concerted motion of all ligands with the symmetric vibration in the dimer either in-phase (mode E) or out-of-phase (D) with the other ligands. A mode analogous to the symmetric “breathing” mode E appears in the simulated spectra of all tetrahedral *n* = 4 complexes, but in this case it gains oscillator strength from the lower symmetry arising from the fifth ligand.

The lowest energy calculated structures (and, thus, spectra) of Ir(NO)_*n*_^+^ are very similar to those of the Co(NO)_*n*_^+^ complexes (Figure 4). The agreement between simulated and experimental spectra is not quite as good for the Rh(NO)_*n*_^+^ complexes, but the dimer motif is almost certainly signified by the band around 1700 cm^–1^. No single isomer provides as convincing a fit as that for the Co(NO)_5_^+^ complex in Figure 5. In particular, the most intense feature at 1790 cm^–1^ is missing from most simulated spectra. However, even a small frequency shift in a single band (for example, in the spectra of structures Rh5I or Rh5 V) would recover it.

**Figure 6:**
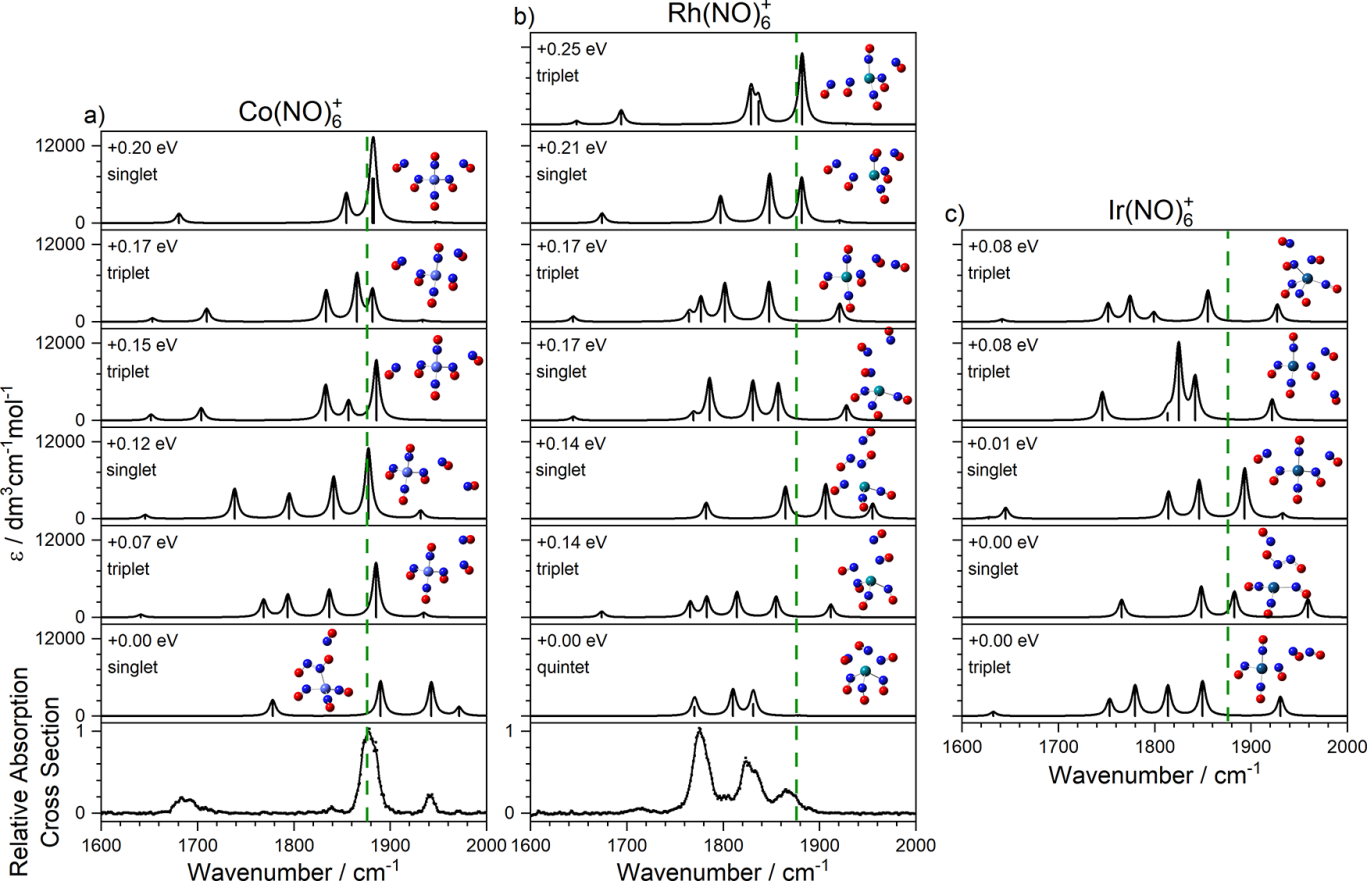
Comparison of experimental and simulated spectra of low-lying isomers for (a) Co(NO)_6_^+^, (b) Rh(NO)_6_^+^, and (c) Ir(NO)_6_^+^. Green dashed lines indicate the wavenumber of the free NO stretch at 1876 cm^–1^.^40^

**Figure 7:**
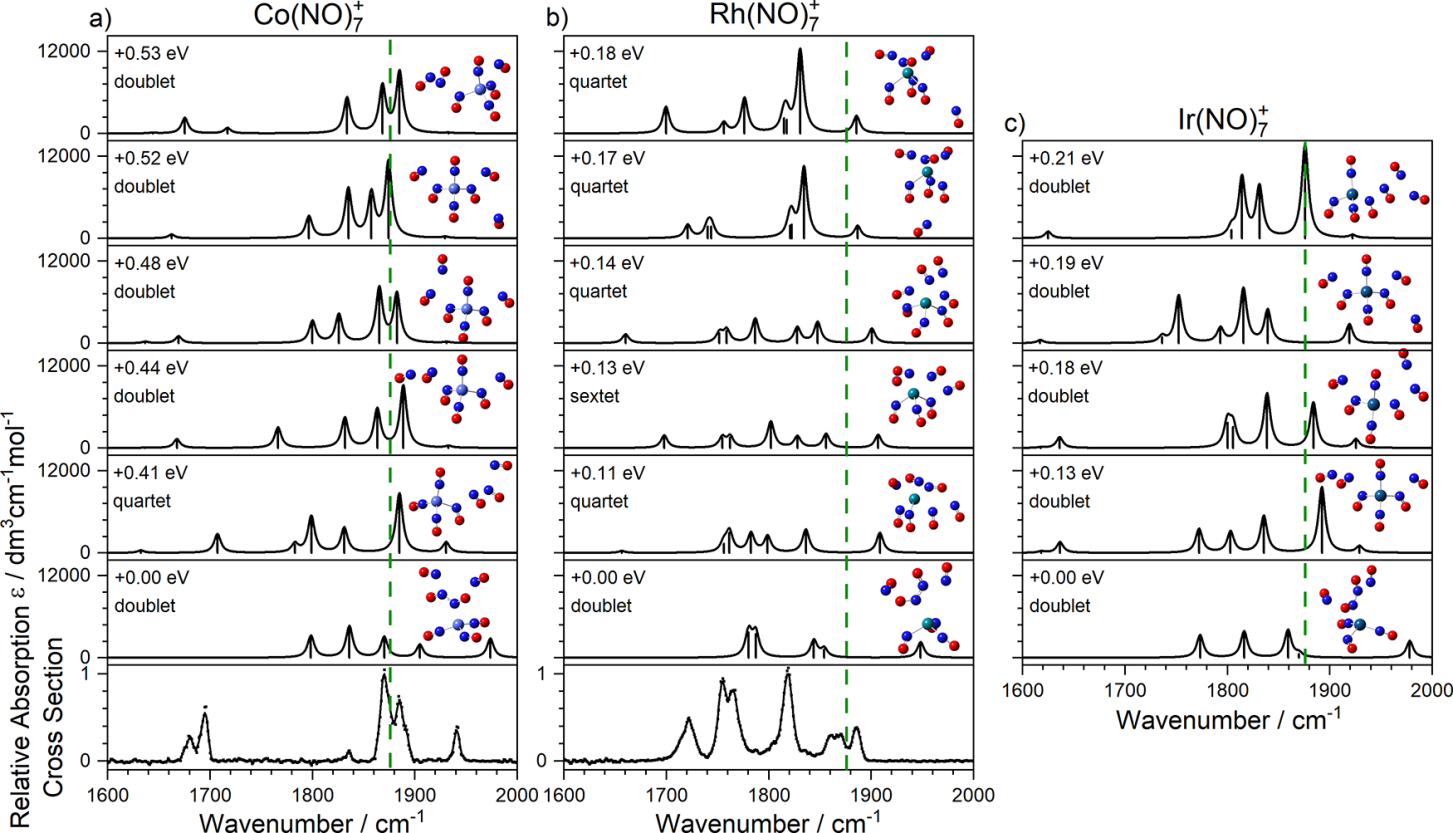
Comparison of experimental and simulated spectra of low-lying isomers for (a) Co(NO)_7_^+^, (b) Rh(NO)_7_^+^, and (c) Ir(NO)_7_^+^. Green dashed lines indicate the wavenumber of the free NO stretch at 1876 cm^–1^.^40^

The spectra of the Co(NO)_*n*_^+^ complexes do not change markedly as sixth and seventh ligands bind, with the exception of the dimer band around 1680 cm^–1^ which splits into (at least) two bands as more dimer moieties form. Figures 6 and 7 contain the comparison of experimental and simulated spectra for energetically low-lying structures of *n* = 6, 7 complexes, respectively. The spectra of the Rh(NO)_*n*_^+^ complexes continue more significant evolution with additional ligands. The trend for the overall spectrum to shift further red of the free NO stretch continues, and more low-lying electronic states are predicted than for Co^+^ or Ir^+^ complexes. The origin of the increased red-shift observed in the spectra of Rh(NO)_*n*_^+^ complexes may lie in the increased propensity for nonlinear (one-electron) binding with Rh^+^. As a result, the addition of more ligands leads to more effective back-bonding into the NO π* system. This is consistent with the smaller spectral shifts and reduced intensity of the dimer bands in the Rh(NO)_*n*_^+^ complexes. Despite the apparent simplicity of the spectra observed, however, it is difficult to make unambiguous assignments beyond the presence of dimer structures, and it seems likely that more than one isomer and/or electronic state is present.

### D Different Dissociation Channels

The spectra shown above in Figures 2–7 were all recorded in the daughter fragment channel corresponding to the loss of a single NO ligand. In some cases, especially for larger complexes, weaker additional fragment channels were observed which provide additional information on the structure of the complex. At a basic level, the degree of fragmentation observed provides additional information on coordination shells with the Co(NO)_*n*_^*+*^ (*n* > 6) showing loss of two ligands consistent with a first coordination shell of 4 (see Supporting Information).

**Figure 8:**
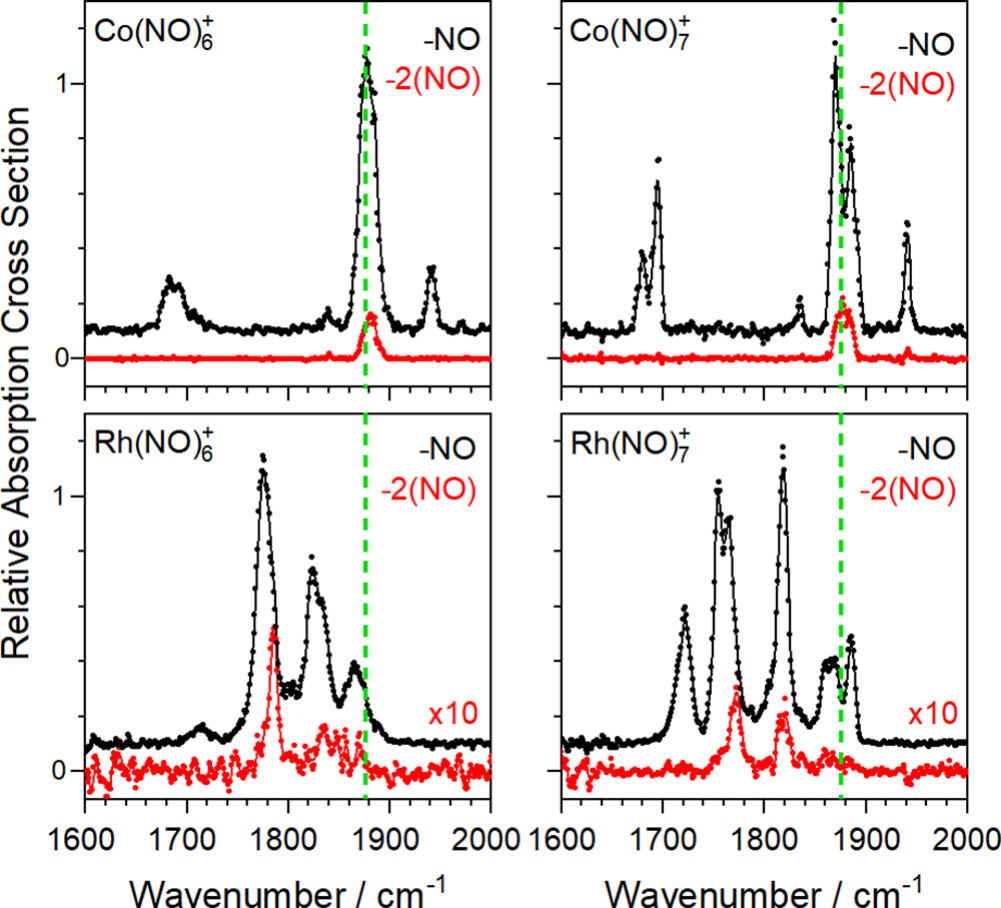
IR-PD spectra of Co(NO)_*n*_^+^ (*n* = 6–7) (upper panels) and Rh(NO)_*n*_^+^ (*n* = 6–7) (lower panels) species, recorded in different fragmentation channels: −NO (black line), −2(NO) (red line). Spectra are given in terms of normalized absorption cross-sections for the −NO channel (black line), and the other channel is scaled accordingly as shown. The NO loss channel spectrum data is shown offset vertically for clarity. Green dashed lines indicate the wavenumber of the free NO stretch at 1876 cm^–1^.^40^

Further structural information is provided by the spectra recorded in the −2(NO) (*i.e*., double ligand loss) fragmentation channels for Co(NO)_*n*_^+^ (*n* = 6, 7) and Rh(NO)_*n*_^+^ (*n* = 6, 7) as shown in Figure 8. In the Co(NO)_*n*_^+^ spectra at least some components of the strong bands in the central (1750–1900 cm^–1^) spectral region appear in both the single, −(NO), and the double, −2(NO), loss channel. The same is true in the Rh(NO)_*n*_^+^ spectra though some of the bands are noticeably narrower and slightly blue-shifted in the −2(NO) channel. It is possible that only one spectral component in a blended band is observed in the double-loss channel. In all cases, however, the peripheral bands, while clear in the −NO loss channel, are essentially absent in the −2(NO) spectra. We interpret this as further confirmation that the outer spectral bands in the spectrum (especially the low frequency band) arise from the dimer moiety (see Figure 5). In these bands, once a first photon is absorbed, a ligand from the outer coordination shell is lost and, with it, crucially the chromophore itself. As a result, no subsequent photon can be absorbed at the same wavenumber to remove a second ligand. These results strongly suggest that the dimer (NO)_2_ moiety itself is not lost as an intact entity but rather the −2(NO) channel comprises two sequential single loss processes. This is consistent with the strength of binding direct to the metal center exceeding the one-photon energy. By contrast, the more central IR modes, which do not involve motion of the dimer (See Figure 5), can absorb more than one photon, leading to the loss of two ligands. In this way, differences in the spectra recorded in different fragment mass channels provide exquisite detail on the nature of the vibrational mode excited.

## 4 Conclusions

A comparison of infrared action spectra with simulated spectra of calculated low energy isomers has revealed the structures of M(NO)_*n*_^*+*^ (M = Co, Rh, Ir) ion–molecule complexes. Notwithstanding the difficulties of applying DFT to multiple open-shell ligands, some clear structural features are observed, including evidence of complete coordination shells. A mix of linear and nonlinear ligand binding motifs is also observed, often within the same complex, and a range of energetically low-lying isomers and/or electronic states is found for all complexes with *n* ≥ 4. The Co(NO)_*n*_^+^ and Ir(NO)_*n*_^+^ complexes exhibit similar spectra/structures throughout the size range studied. The spectra of the Rh(NO)_*n*_^+^ complexes appear qualitatively different which may result from an increased propensity for nonlinear (one-electron) ligand binding.

In common with previous studies of gas-phase metal nitrosyl complexes, there is clear evidence for the formation of NO dimers once binding in a second coordination shell occurs at *n* ≥ 5. New spectral features appear that are not present in the spectra of smaller complexes, including a strongly red-shifted band (≤1700 cm^–1^). Further evidence for some of the vibrational mode assignments comes from the difference in the action spectra observed in different fragmentation channels, illustrating the benefits of parent ion mass-selection.

## Supporting Information Available

Additional time-of-flight spectra, computational structure details, comparison of experimental and simulated spectra for additional complex sizes, photofragment time-of-flight spectra, relative energy of electronic states (PDF)
