# Supplementary material for: An Infrared Study of Gas-Phase Metal Nitrosyl Ion–Molecule Complexes
Source: J Phys Chem A. 2022 Dec 8;126(50):9414–22. doi: 10.1021/acs.jpca.2c07228 (PMC9791661; doi:10.1021/acs.jpca.2c07228)
Supplement: Supplementary file 1 — jp2c07228_si_001.pdf [file jp2c07228_si_001.pdf]

An Infrared Study of Gas-Phase Metal Nitrosyl Ion-Molecule Complexes

*Gabriele Meizyte, Philip A. J. Pearcy, Peter D. Watson, Edward I. Brewer, Alice E. Green, Matthew Doll, Olga A. Duda, and Stuart R. Mackenzie**

Department of Chemistry, University of Oxford, Physical and Theoretical Chemistry Laboratory, South Parks Road, Oxford, United Kingdom, OX1 3QZ

Supporting Information

*stuart.mackenzie@chem.ox.ac.uk

Contents

1. Time-of-Flight Mass Spectra 3

1.1. Rhodium (IR off) 3

1.2. Iridium (IR off) 4

1.3. Cobalt and Rhodium (IR on) photofragment yields 5

2. Calculated Structures 6

3. Coordinates and Geometries of Relevant Isomers 6

3.1. Structures and coordinates of low energy isomers of $Co(NO)_3^+$ 6

3.2. Structures and coordinates of low energy isomers of $Rh(NO)_3^+$ 6

3.3. Structures and coordinates of low energy isomers of $Ir(NO)_3^+$ 7

3.4. Structures and coordinates of low energy isomers of $Co(NO)_4^+$ 8

3.5. Structures and coordinates of low energy isomers of $Rh(NO)_4^+$ 9

3.6. Structures and coordinates of low energy isomers of $Ir(NO)_4^+$ 10

3.7. Structures and coordinates of low energy isomers of $Co(NO)_5^+$ 11

3.8. Structures and coordinates of low energy isomers of $Rh(NO)_5^+$ 13

3.9. Structures and coordinates of low energy isomers of $Ir(NO)_5^+$ 15

4. Comparison of Experimental and Computational Spectra of Low-Lying Isomers 17

4.1. $M(NO)_3^+$ 17

4.2. $M(NO)_4^+$ 18

4.3. $M(NO)_6^+$ 19

4.4. $M(NO)_7^+$ 20

5. Binding Energies 21

6. Low-lying Electronic States 22

Time-of-Flight Mass Spectra

Rhodium (IR off)

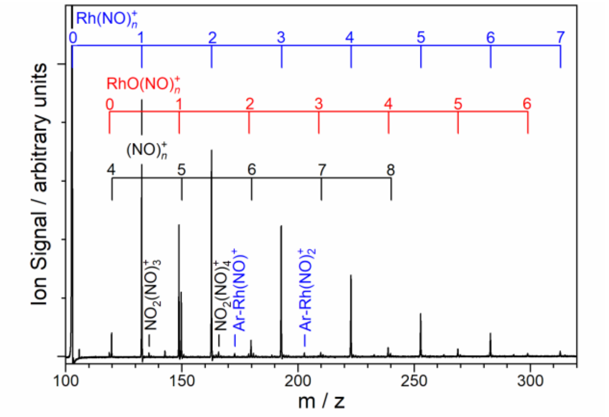

Figure S1. Time-of-flight mass spectrum produced upon ablating the Rh metal target in presence of NO in Ar gas mix (1.5%).

Iridium (IR off)

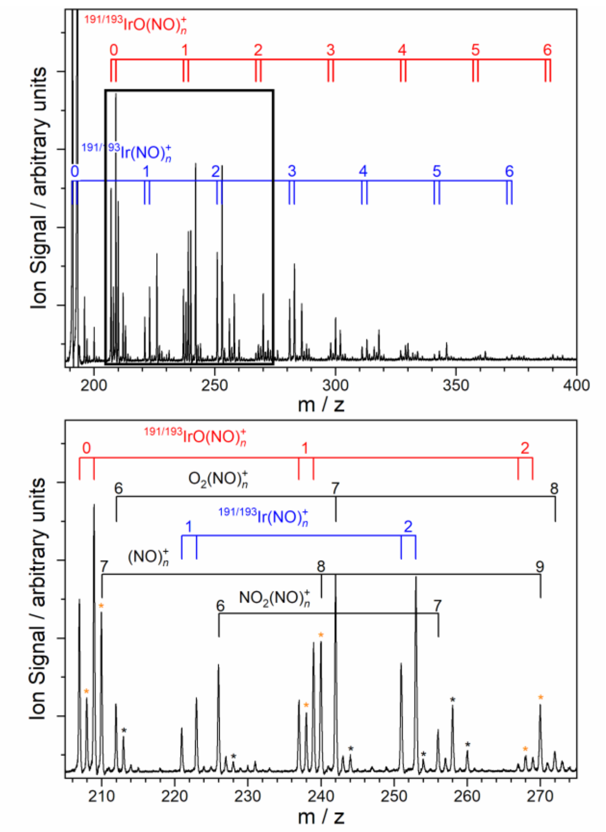

Figure S2. Time-of-flight mass spectrum produced upon ablating the Ir metal target in presence of NO in Ar gas mix (1.5%). The bottom panel shows a zoomed in region indicated in the top panel. Orange and black asterisks mark peaks that arise from possible water contamination and pure $(NO)_n^+$-containing species, respectively.

Cobalt and Rhodium (IR on) photofragment yields

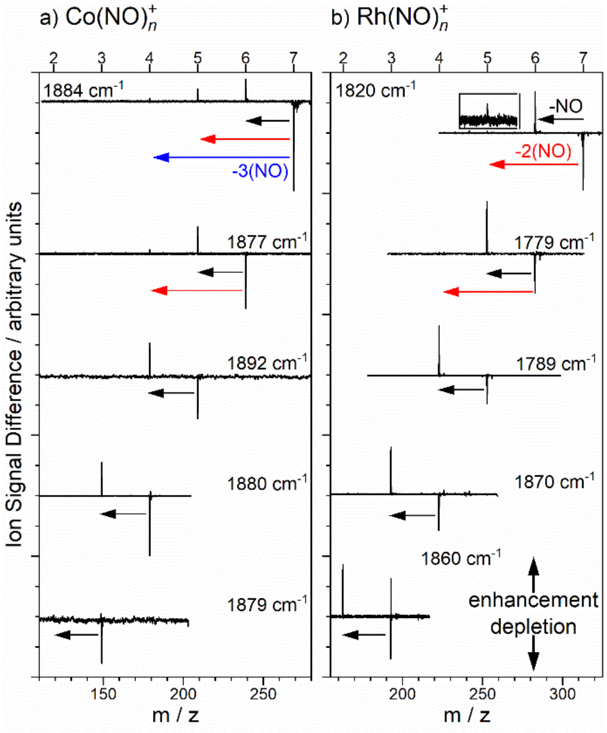

Figure S3. Time-of-flight mass spectra for a) $Co(NO)_n^+$ and b) $Rh(NO)_n^+$ (n = 3-7) species recorded as averages of 1000 shots with IR laser on and at maximum resonance wavenumbers, as indicated. Enhancement and depletion correspond to the peaks pointing up and down, respectively. The -NO, -2(NO), and -3(NO) loss channels are specified by black, red, and blue arrows, respectively.

Calculated Structures

Coordinates and Geometries of Relevant Isomers

Structures and coordinates of low energy isomers of $Co(NO)_3^+$

Co3I

O	-0.44988700	2.79369500	0.00163900
N	-0.27111800	1.68513400	-0.00048200
O	-2.19558400	-1.78561400	0.00159700
N	-1.32405900	-1.07758600	-0.00039900
Co	0.00018600	-0.00016800	-0.00115800
O	2.64475000	-1.00714300	0.00134700
N	1.59528100	-0.60797400	0.00011100

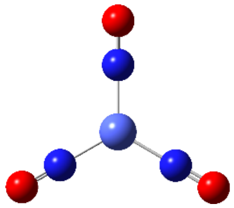

Co3II

O	-1.46144900	2.36838800	0.26452200
N	-0.81466900	1.47982500	0.01111600
O	-1.48202800	-2.35616800	0.26478400
N	-0.82740300	-1.47339600	0.01126700
Co	-0.03180400	-0.00021400	-0.22374100
O	1.94302400	-0.00758500	-0.17746900
N	2.90812100	-0.01090300	0.43851700

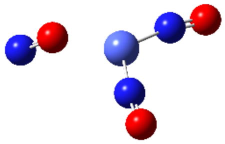

Structures and coordinates of low energy isomers of $Rh(NO)_3^+$

Rh3I

O	0.76498400	2.51177400	0.78536200
N	0.52360400	1.72222700	0.02149800
Rh	-0.00035200	-0.00039100	-0.42934800
O	-2.55820400	-0.59353200	0.78590700
N	-1.75314000	-0.40763400	0.02273000
O	1.79478500	-1.91687000	0.78542200
N	1.23001400	-1.31364700	0.02250800

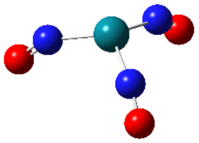

Rh3II

O	-1.87305300	-2.08367900	0.23510700
N	-0.95955400	-1.46502300	0.03434700
Rh	0.03191200	-0.00002200	-0.13601300
O	-1.87308700	2.08455600	0.23407900
N	-0.96055800	1.46394900	0.03477100
O	2.39735700	-0.00173600	-0.22664500
N	3.25642900	0.00219800	0.52806100

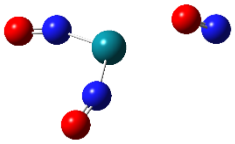

Structures and coordinates of low energy isomers of $Ir(NO)_3^+$

Ir3I

N	0.17008300	-1.82057900	-0.00013800
O	0.27560600	-2.94147900	0.00154900
N	1.49247200	1.05737400	0.00063800
O	2.41173500	1.70714800	0.00102900
N	-1.66241400	0.76261700	0.00038700
O	-2.68601000	1.23122500	0.00121400
Ir	-0.00015100	0.00037600	-0.00047500

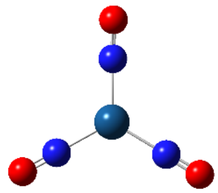

Ir3II

N	0.90474300	-1.66006600	-0.00133600
O	1.95691900	-2.08340300	0.00324000
N	0.36005600	2.01791900	0.00406100
O	1.46659000	1.59952200	0.00021800
N	-1.73704400	-0.31286600	0.00094900
O	-2.79419400	-0.70140300	0.00201700
Ir	-0.02245200	0.11905700	-0.00090300

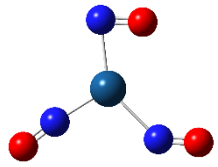

Structures and coordinates of low energy isomers of $Co(NO)_4^+$

<u>Co4I</u>

O	-1.63139700	1.64795500	1.43632900
N	-0.63338100	1.13040900	1.29194400
O	-2.20025700	-1.58503700	-1.04232000
N	-1.36204200	-0.94430000	-0.64255100
O	1.63135800	1.64813500	-1.43625700
N	0.63317200	1.13100900	-1.29153700
Co	0.00001700	-0.13397300	-0.00002600
O	2.20037800	-1.58505100	1.04210800
N	1.36209300	-0.94436800	0.64240400

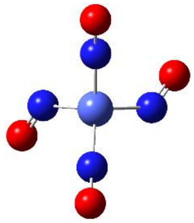

<u>Co4II</u>

O	-1.50109500	-1.89361500	-0.00031700
N	-2.91739600	0.64095400	0.00133000
O	-3.71659500	-0.13951300	0.00093500
N	-0.77888400	-1.01720400	-0.00061900
O	0.75769800	2.83434500	0.00079800
N	0.63556500	1.71043300	-0.00052200
Co	0.63796400	0.00808700	-0.00117400
O	3.13504400	-1.28764600	0.00263600
N	2.11422500	-0.80945600	-0.00029100

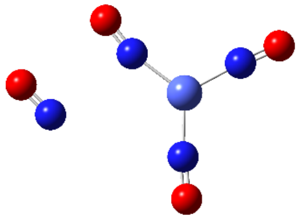

<u>Co4III</u>

O	-2.04684800	-0.00499000	1.68418200
N	-0.92515100	-0.00211700	1.70362300
O	-0.72939000	-2.42657100	-1.20823800
N	-0.38160200	-1.45484300	-0.75971500
O	-0.74675100	2.42004400	-1.21043300
N	-0.39152900	1.45136800	-0.76114800
Co	0.17931900	0.00051000	-0.14295300
O	2.85920000	0.00962700	0.68316000
N	1.76524000	0.00578500	0.42729000

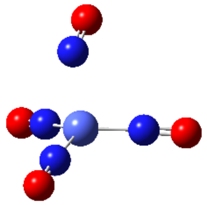

Structures and coordinates of low energy isomers of $Rh(NO)_4^+$

<u>Rh4I</u>

O	-0.75761100	-0.00427500	2.48260600
N	0.06466700	-0.00246600	1.70656100
Rh	-0.05611800	-0.00011900	-0.23144200
O	1.71503100	2.33662800	-0.08601000
N	1.23265400	1.48643700	-0.65710700
O	-2.95317800	-0.00215800	-0.85610800
N	-1.86504700	-0.00207500	-0.55833300
O	1.72443000	-2.33035300	-0.09077500
N	1.23857500	-1.48094900	-0.66009400

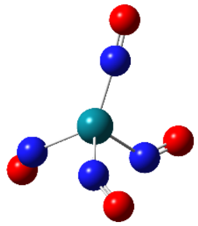

<u>Rh4II</u>

N	0.25454400	-1.11286400	1.56468700
O	1.26882300	-1.35041900	2.00559300
Rh	-0.17843600	0.16017500	-0.00191600
N	-2.06053900	0.17358000	-0.00239700
N	1.10874600	1.41791300	-0.01496500
N	0.25590200	-1.14670700	-1.54027900
O	-3.17377400	0.35170600	-0.00409900
O	2.02445300	2.07627500	-0.02160100
O	1.27038000	-1.39397800	-1.97527900

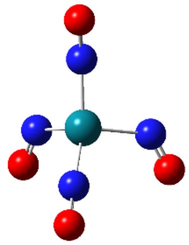

<u>Rh4III</u>

O	1.87820900	-0.99765000	-0.05057700
N	0.89762500	-0.66287900	-0.51855700
Rh	-0.86279200	-0.01265400	-0.43501800
O	-1.07911200	2.60897300	0.76199900
N	-1.05420000	1.75427800	0.02312400
O	-2.56570800	-1.64412500	1.23142000
N	-2.06734700	-1.15421400	0.34436200
O	4.96393500	-0.22879300	0.46925700
N	4.11635800	0.44312800	0.19093000

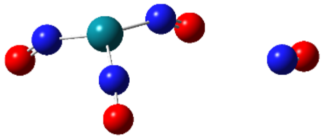

Structures and coordinates of low energy isomers of $Ir(NO)_4^+$

Ir4I

N	0.10139500	1.54418300	1.21785400
O	1.04512100	2.12644500	1.45410800
Ir	-0.10594200	-0.00019000	-0.15171200
N	-1.94830200	-0.00004400	-0.34599300
N	1.38105600	-0.00152200	-1.15038500
N	0.10108900	-1.54151200	1.22080300
O	-3.07343500	-0.00045700	-0.49551000
O	2.32236800	-0.00197100	-1.78130800
O	1.04481000	-2.12315800	1.45844400

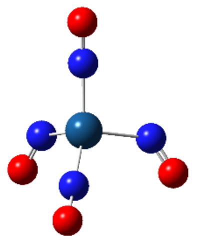

Ir4II

N	-4.50890900	0.45895900	0.00120200
O	-5.35680900	-0.25977400	-0.00086900
Ir	0.60367600	-0.00587400	-0.00006600
N	1.00389400	1.76234100	-0.00006600
N	-1.14701600	-0.58409500	0.00004200
N	1.94403500	-1.22674100	0.00001400
O	1.31792200	2.85149800	0.00003700
O	-2.22653000	-0.95481900	0.00013700
O	2.82453200	-1.93951900	0.00028300

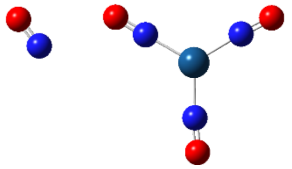

Ir4III

N	-0.81344300	0.00256200	-2.04306500
O	-1.92739200	0.00726800	-2.13296600
Ir	0.12195900	-0.00048200	0.16145400
N	1.86331500	-0.01156100	-0.40165400
N	-0.58925000	1.58593500	0.71873600
N	-0.60893800	-1.57676600	0.72224500
O	2.95106600	-0.01821800	-0.71213500
O	-1.01776700	2.56964100	1.08198000
O	-1.04998100	-2.55420400	1.08739600

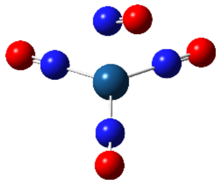

Structures and coordinates of low energy isomers of $Co(NO)_5^+$

Co5I

O	-1.18823400	-1.82279600	-1.81745300
N	-1.11141100	-0.71143900	-1.59495000
O	-1.96913900	-1.00758500	2.16228600
N	-1.45553700	-0.50736900	1.28895800
O	1.58949300	-1.49300900	0.01810900
N	1.19774100	-0.40736900	0.01000000
Co	-0.63960500	0.19885300	0.00781100
O	-0.73932900	3.02037500	-0.35748100
N	-0.64079800	1.89305500	-0.32682800
O	3.66350200	-0.17684500	0.29449900
N	2.92700400	0.65738400	0.24988200

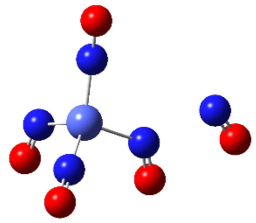

Co5II

O	0.46856500	-2.08295300	1.67332000
N	0.37664100	-0.97022600	1.45550800
O	2.99960900	-1.16074400	-1.40975700
N	2.11278500	-0.71987300	-0.86480300
O	-1.75924700	0.50564000	-0.93290000
N	-0.75285000	-0.02700900	-1.04232800
Co	0.88671600	0.10698200	-0.01115200
O	1.21609700	2.80252700	0.81683700
N	1.01648200	1.73374800	0.50229100
O	-4.85466900	0.04118300	-0.24509600
N	-3.96793900	-0.55003300	0.10388400

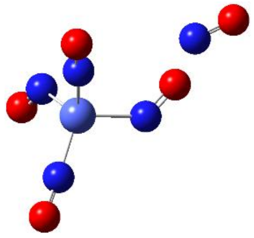

Co5III

O	1.65228100	-1.31536000	2.14206900
N	1.42959500	-0.33121200	1.61956000
O	1.27417700	-2.02146300	-1.89572900
N	1.02486100	-1.22628400	-1.13097700
O	-1.76577400	0.78872200	0.99119800
N	-1.15114100	0.00538800	0.42369000
Co	0.70993300	0.08558300	-0.09306100
O	1.32838000	2.75999500	-0.80428800
N	1.06528800	1.71527400	-0.45878700
O	-3.45448300	-0.70188100	-0.55583100
N	-4.00358100	0.06671400	0.04555600

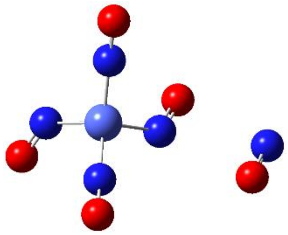

Co5IV

N	-4.23306700	0.53057300	-0.02975300
O	-5.19593800	-0.04423500	0.04663800
N	1.72195900	-1.32852500	-0.95783600
O	2.81986900	-1.59998700	-0.84826100
Co	0.64574300	-0.08557400	0.03877500
N	-0.97166600	-0.67476300	-0.07908900
N	1.41114000	0.29985100	1.48264000
N	0.77888000	1.38400500	-1.18981100
O	-2.02868900	-1.06889400	0.05416500
O	1.87809400	0.53715900	2.48453000
O	1.47844300	2.28002200	-1.19082100

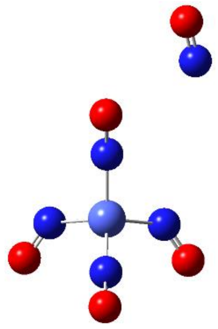

Co5V

O	-0.25831200	2.88110200	-0.47308600
N	-0.13123400	1.76371500	-0.34859800
O	0.32496700	-1.14959900	2.40551500
N	-0.26374400	-0.78927600	1.49776800
O	2.72789700	0.59705400	0.54674200
N	1.99149700	-0.05852100	-0.01655400
Co	0.07670100	0.07993200	-0.09669500
O	-3.07732700	-0.24369100	0.46890900
N	-2.39053300	-0.10794300	-0.41349000
O	0.73764600	-1.91132300	-2.01268400
N	-0.02168700	-1.31461800	-1.41518300

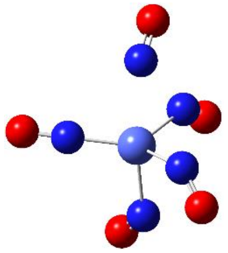

Structures and coordinates of low energy isomers of $Rh(NO)_5^+$

<u>Rh5I</u>

O	0.41025000	-0.03132400	-2.62344400
N	-0.27662200	-0.23874800	-1.74279500
Rh	0.01331800	0.02306300	0.17864800
O	1.41851200	-2.58452600	0.81959700
N	0.43495900	-2.04182900	0.67443400
O	2.54783100	1.31096200	0.11696100
N	1.98265900	0.32397700	0.08622700
O	-0.84059300	2.78228100	0.62405900
N	-0.53953800	1.70009500	0.78266200
O	-3.06481500	-0.66558000	-0.22118200
N	-2.22556900	-0.81954200	0.51846100

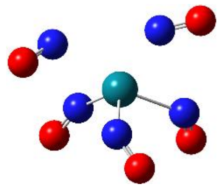

<u>Rh5II</u>

O	-1.40122000	-1.75479700	-1.97338100
N	-1.11486700	-0.68171500	-1.74419900
O	-1.89933900	-1.23501800	2.17483700
N	-1.46706000	-0.59236400	1.35189900
O	1.76440700	-1.58615000	0.13668500
N	1.40598200	-0.49982200	0.00373000
Rh	-0.56758700	0.18845200	0.01777700
O	-0.53909500	3.13989900	-0.11382100
N	-0.44341200	2.02859400	-0.29963900
O	3.89664300	-0.31422000	0.20769600
N	3.18653500	0.53415800	0.08019500

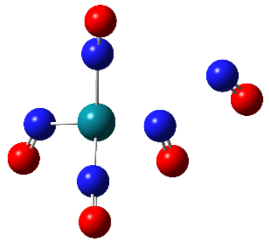

<u>Rh5III</u>

O	-1.68727700	-0.01317100	1.13614900
N	-0.70073800	0.51130300	0.90695100
Rh	0.83170600	-0.14770400	-0.16144400
O	0.04038800	-2.90970700	-0.87423600
N	0.33705900	-1.85650400	-0.58507300
O	2.38935000	0.60543800	2.19343800
N	2.29968800	0.42470700	1.07723300
O	-4.87984800	0.17757900	0.49331800
N	-4.00100600	0.51078100	-0.11962900
O	0.35713300	2.13779300	-1.90516900
N	1.03860900	1.36160000	-1.43420100

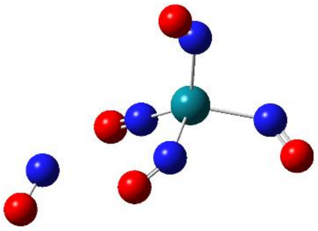

Rh5IV

N	4.55161300	0.57266200	-0.00756700
O	5.51828700	0.00893100	-0.11885700
N	-1.40514900	-1.31457500	1.26501600
O	-2.50708800	-1.53530800	1.41768200
Rh	-0.54879700	-0.09133100	-0.15247600
N	1.23766200	-0.61784500	-0.11774200
N	-1.81487700	0.15996900	-1.40508400
N	-0.49160000	1.62963700	1.02086300
O	2.29230700	-1.03106400	-0.17517000
O	-2.68559300	0.33997200	-2.10318000
O	-1.34887100	2.35508700	1.17615100

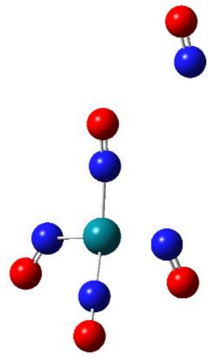

Rh5V

O	2.96231000	0.24414300	-0.45774500
N	2.06800300	-0.42918500	-0.28352400
Rh	0.19825700	0.07156700	0.00638100
O	-0.24304900	-1.84493300	-2.16190100
N	-0.62099600	-1.22019500	-1.29404000
O	0.18076000	3.03485700	-0.06448300
N	0.21878100	1.90562600	-0.04542100
O	0.57793100	-1.57243500	2.39025100
N	-0.11895500	-1.08624700	1.63951200
O	-2.80940500	0.51690500	0.40727900
N	-3.58539900	-0.06268400	-0.18714800

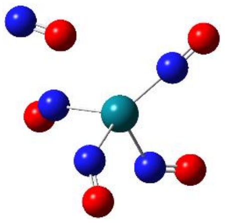

Structures and coordinates of low energy isomers of $Ir(NO)_5^+$

Ir5I

N	1.03881100	-0.04620000	1.83688300
O	1.56882100	-0.93799700	2.30334800
N	-3.31942700	0.27459000	-0.00890600
O	-3.97637700	-0.62096700	0.00098600
Ir	0.43182000	0.15959100	-0.11445000
N	-1.47877700	-0.63686200	0.08578000
N	0.11380800	1.96846600	-0.17913100
N	1.41773800	-1.01517500	-1.03454200
O	-1.76863200	-1.75612600	0.09678700
O	-0.03391900	3.09106700	-0.28681700
O	2.00320300	-1.78875600	-1.62529800

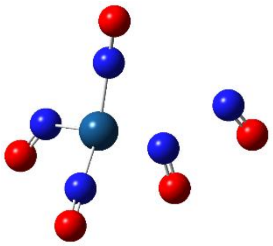

Ir5II

O	0.44990600	-1.45508600	2.31659700
N	0.23312100	-0.44833700	1.82594600
O	2.51822900	-1.88744400	-1.15080700
N	1.81266900	-1.10707400	-0.72158400
O	-2.08425600	0.01862900	-1.03010300
N	-1.07682300	-0.52687400	-1.03827500
Ir	0.66175100	0.13659300	-0.06637400
O	0.32285400	3.06133700	0.26345100
N	0.44756800	1.94054600	0.10785400
O	-5.12119300	-0.28970000	-0.18623000
N	-4.22212600	-0.72962200	0.31284400

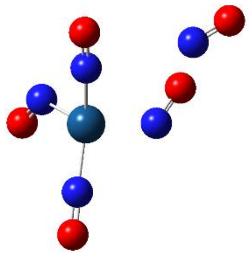

Ir5III

O	1.22323800	-1.18875700	2.38568400
N	1.18049000	-0.22276800	1.78596100
O	0.48403200	-2.69250800	-1.33956100
N	0.54100200	-1.65691800	-0.87241700
O	-1.98049600	1.21163600	0.54604300
N	-1.48829200	0.22578600	0.21896800
Ir	0.54357300	0.00567900	-0.15383200
O	1.55040100	2.74128200	-0.67013100
N	1.16813600	1.68763500	-0.47751800
O	-3.94064600	-0.54302000	-0.16705800
N	-4.33667100	0.44249900	0.17432800

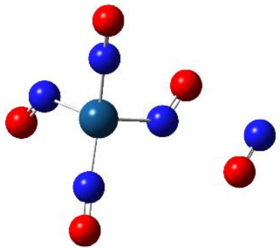

Ir5IV

N	4.63389400	0.59879800	0.12680200
O	5.53859800	-0.01110300	-0.12863100
N	-1.14777700	-1.47755000	1.16199900
O	-2.22753800	-1.78731800	1.34389000
Ir	-0.45037600	-0.06658900	-0.15581900
N	1.31647800	-0.57490500	-0.29674500
N	-1.85275000	0.41804400	-1.15661800
N	-0.23520100	1.44749200	1.27469200
O	2.40867900	-0.90152900	-0.39895900
O	-2.74178400	0.74019400	-1.78734300
O	-1.01840300	2.24028000	1.49944000

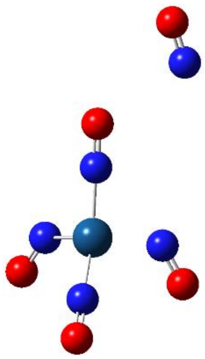

Ir5V

N	1.96528500	-0.09919800	0.38537100
N	-0.18502200	1.86217900	0.00515300
N	0.16939800	-1.21519000	-1.62108100
O	2.73094900	0.73081600	0.55297900
O	-0.25470200	2.99643700	0.03222200
O	1.04892500	-1.50612700	-2.28076500
Ir	0.03039400	0.07422500	-0.01908200
O	-3.19909200	-0.11217600	0.20414100
N	-2.41302600	-0.17941700	-0.60308800
O	0.14209100	-1.89239400	2.16318400
N	-0.40602100	-1.43233900	1.27581700

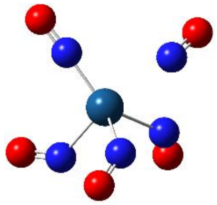

Comparison of Experimental and Computational Spectra of Low-Lying Isomers

$M(NO)_3^+$

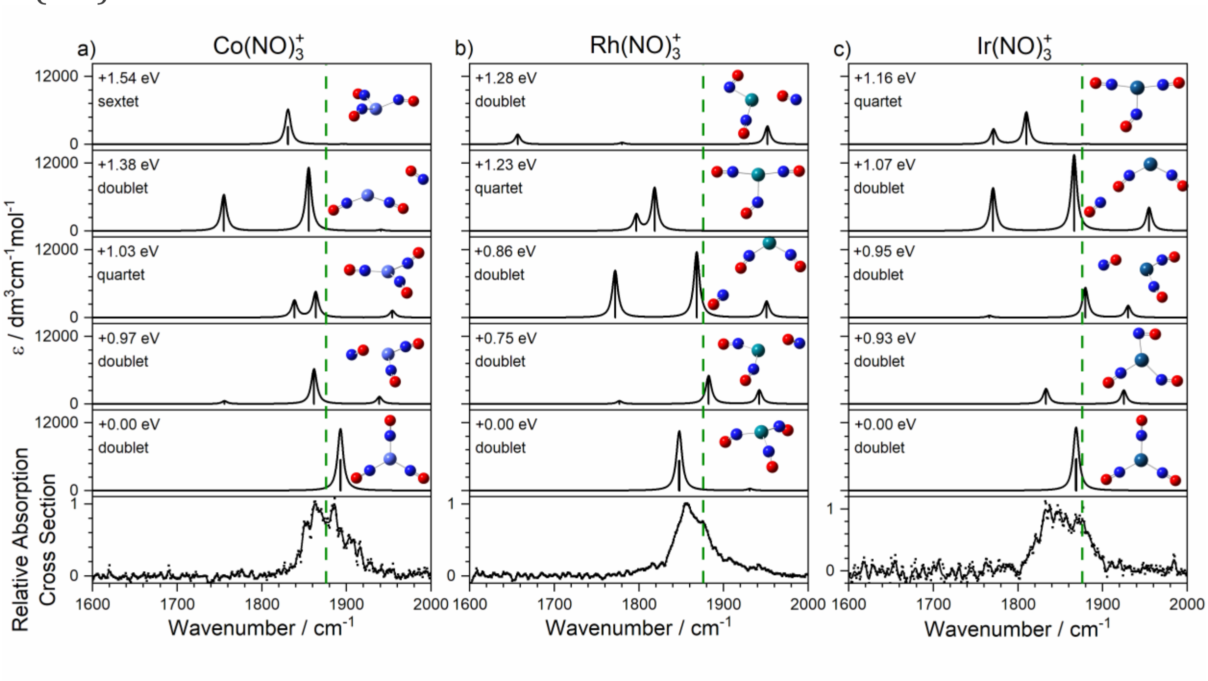

Figure S4. Calculated energetically low-lying isomers of a) $Co(NO)_3^+$, b) $Rh(NO)_3^+$, and c) $Ir(NO)_3^+$.

$M(NO)_4^+$

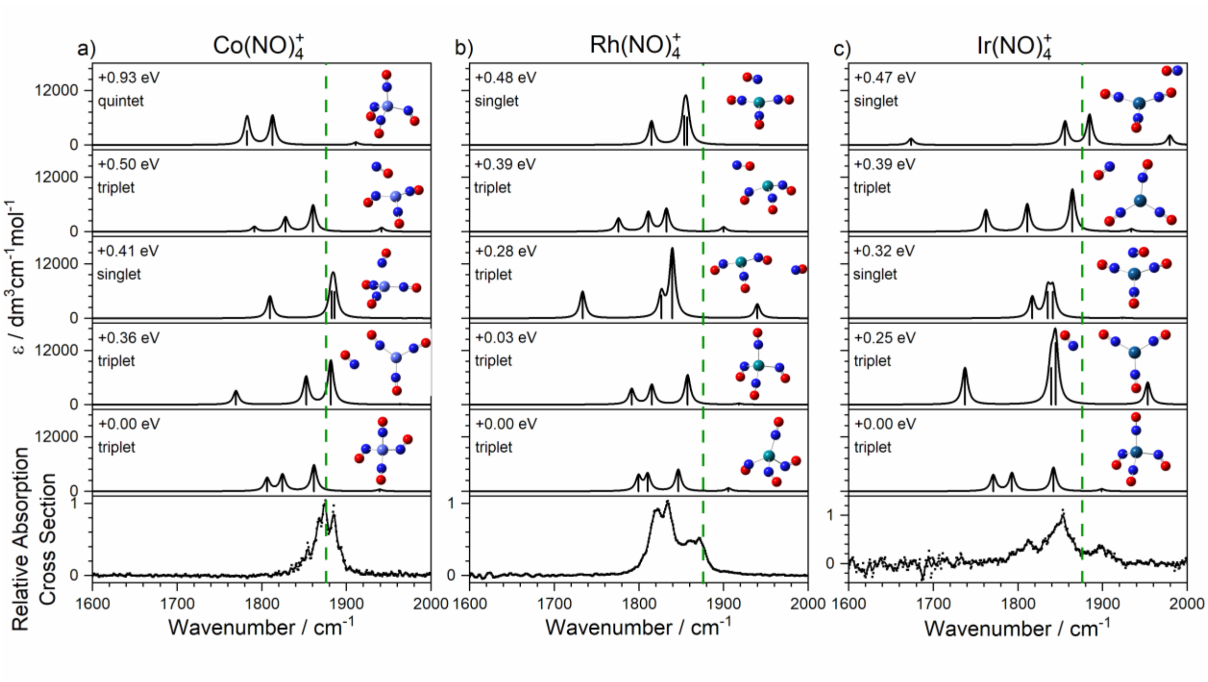

Figure S5. Calculated energetically low-lying isomers of a) $Co(NO)_4^+$, b) $Rh(NO)_4^+$, and c) $Ir(NO)_4^+$.

$M(NO)_6^+$

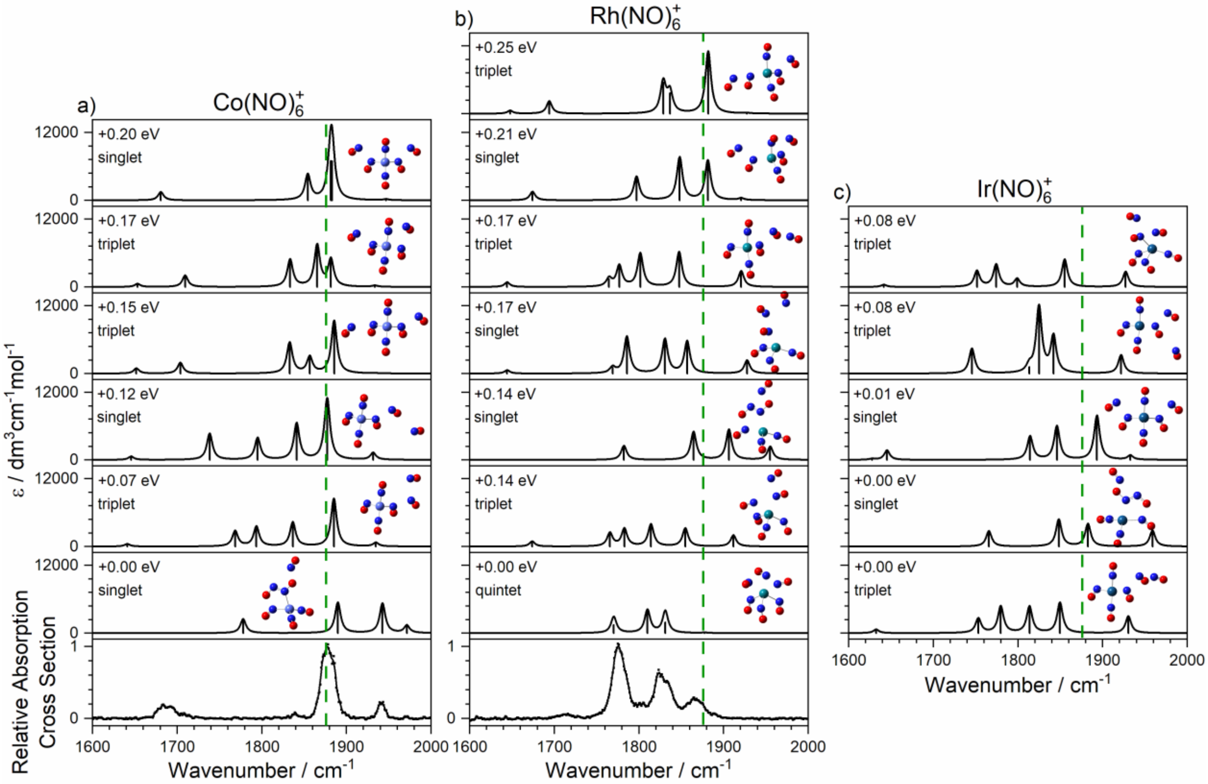

Figure S6. Calculated energetically low-lying isomers of a) $Co(NO)_6^+$, b) $Rh(NO)_6^+$, and c) $Ir(NO)_6^+$.

$M(NO)_7^+$

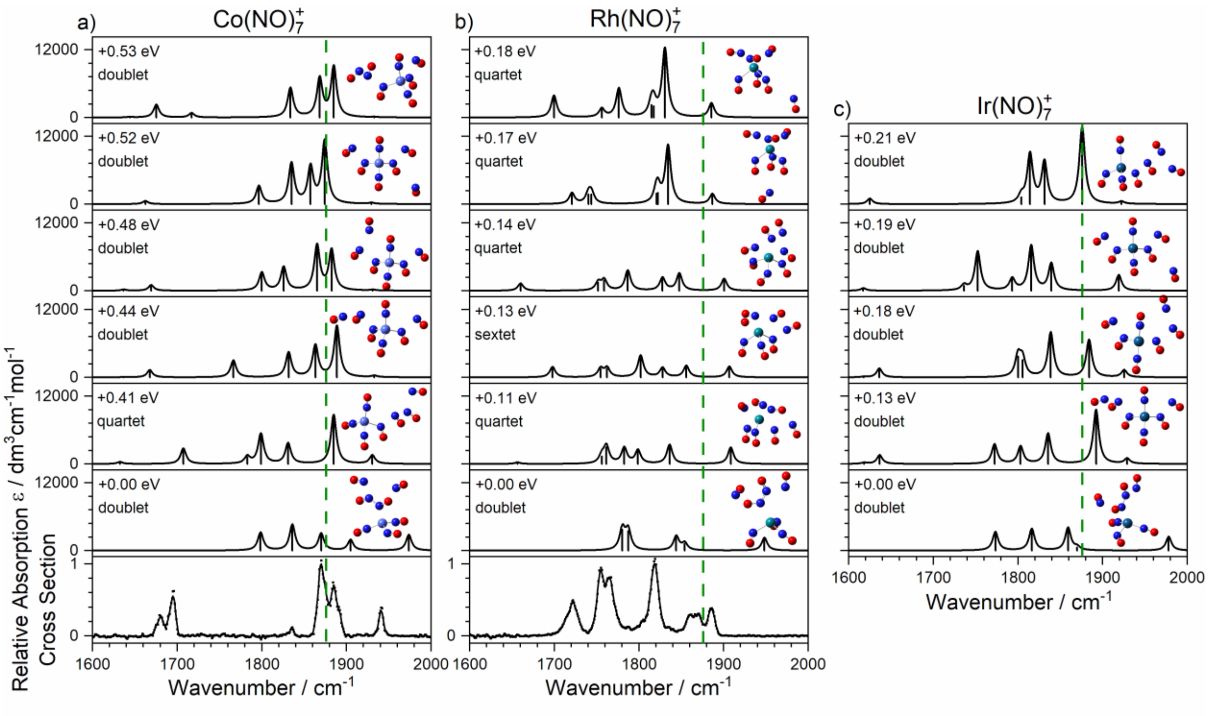

Figure S7. Calculated energetically low-lying isomers of a) $Co(NO)_7^+$, b) $Rh(NO)_7^+$, and c) $Ir(NO)_7^+$.

Binding Energies

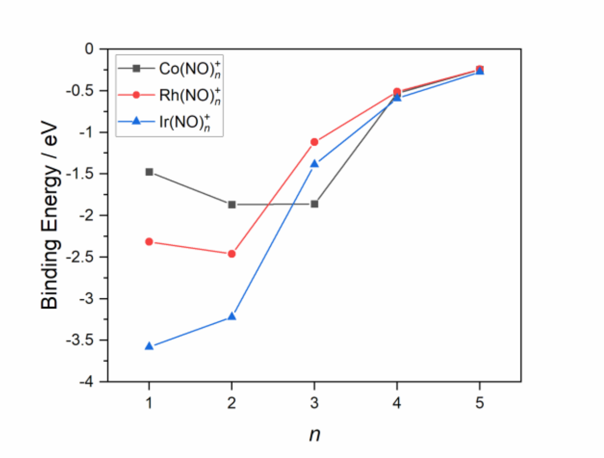

Figure S8. Calculated binding energies for the lowest energy $Co(NO)_n^+$ (black), $Rh(NO)_n^+$ (red), and $Ir(NO)_n^+$ (blue) (n = 1-5) molecularly-bound structures.

Calculated as $E_{bind} = E[M(NO)_n^+] – (E[M(NO)_{n-1}^+] + E[NO])$. Values given in Table S1 below

Table S1. Calculated binding energies (in eV) for the lowest energy $Co(NO)_n^+$, $Rh(NO)_n^+$, and $Ir(NO)_n^+$ (n = 1-5) molecularly-bound structures.

Calculated as $E_{bind} = E[M(NO)_n^+] – (E[M(NO)_{n-1}^+] + E[NO])$.

n	**$Co(NO)_n^+$**	**$Rh(NO)_n^+$**	**$Ir(NO)_n^+$**
**1**	-1.478	-2.315	-3.578
**2**	-1.869	-2.463	-3.221
**3**	-1.863	-1.117	-1.387
**4**	-0.530	-0.512	-0.592
**5**	-0.245	-0.243	-0.275

Low-lying Electronic States

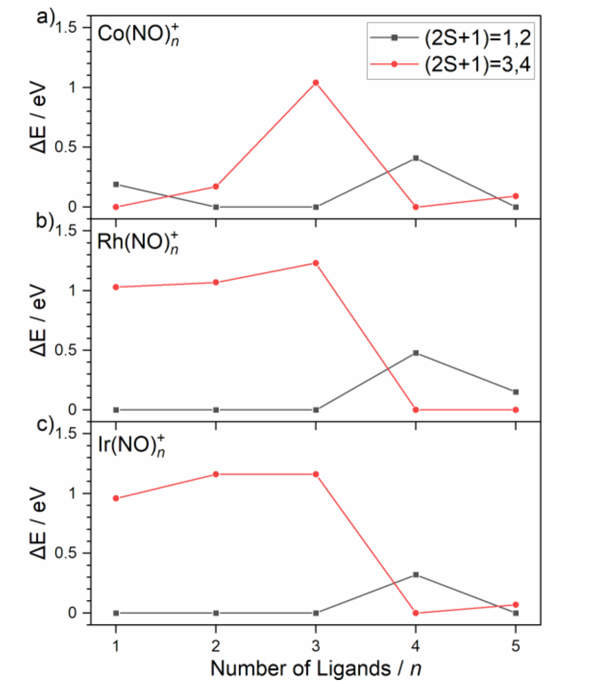

Figure S9. Calculated energies of the lowest energy isomers for a) $Co(NO)_n^+$, b) $Rh(NO)_n^+$, and c) $Ir(NO)_n^+$ (n = 1–5); in black for the lowest spin state (singlet and doublet for even and odd n, respectively) and in red for higher spin state (triplet and quartet for even and odd n, respectively).
